# Trend of *Candida* species profiles and their susceptibility to various antifungals in Jakarta, Indonesia

**DOI:** 10.22034/cmm.2025.345421.1672

**Published:** 2025-02-01

**Authors:** Conny Riana Tjampakasari, Yeva Rosana, Mardiastuti Wahid

**Affiliations:** Department of Clinical Microbiology, Faculty of Medicine, Universitas Indonesia, Indonesia

**Keywords:** Antifungal agents, Antifungal resistance, Antifungal susceptibility, *Candida* species

## Abstract

**Background and Purpose:**

Fungal infections are prevalent in tropical countries, and their etiology depends on various factors, including the underlying cause and accompanying risk factors. Accurate diagnosis through culture and identification is crucial to determine the causative agent, followed by antifungal susceptibility testing to guide appropriate management. *Candida* species are the most commonly found causative agents in fungal infection cases. Given the diverse range of *Candida* causing infections, antifungal treatments vary accordingly. This study aimed to investigate *Candida* species profiles and evaluate their susceptibility to various antifungal agents, providing valuable insights for effective treatment strategies.

**Materials and Methods:**

This study was conducted during 2020-2024, consisting of 2,470 samples of various specimens. Samples were inoculated onto Sabouraud Dextrose Agar for fungal culture, and isolates were subsequently identified and subjected to antifungal susceptibility testing using the Vitek 2 compact automated system.

**Results:**

A total of 799 *Candida* isolates (32.34%) were identified, comprising 21 species. The most prevalent species were *Candida albicans* (44.68%, 357/799), *Candida glabrata* (17.02%, 136/799), and *Candida tropicalis* (16.15%, 129/799). Susceptibility testing of predominant *Candida* species revealed susceptibility rates of 96.06% to fluconazole, 93.51% to voriconazole, 69.84% to caspofungin, 95.64% to micafungin, 95.07% to amphotericin B, and 97.40% to flucytosine. Several uncommon *Candida* species were successfully identified, including *Candida lusitaniae*, *Candida pelliculosa*, *Candida rugosa*, *Candida intermedia*, *Candida kefyr*, *Candida lipolytica*, and *Candida utilis*. These species demonstrated susceptibility to voriconazole, amphotericin B, and flucytosine. However, antifungal susceptibility data were not available for other uncommon species, such as *Candida laurentii*, *Candida famata*, *Candida colliculosa*, *Candida magnoliae*, *Candida sphaerica*, and *Candida* haemulonii.

**Conclusion:**

The trends in *Candida* species profiles and their susceptibility to various antifungal agents over consecutive years revealed variability in both species distribution and susceptibility rates. Understanding the profile and susceptibility of *Candida* species is essential for developing effective treatment strategies.

## Introduction

Mycosis is a disease caused by fungi, including yeast and mold, that can affect various body parts, such as the skin, nails, mouth, throat, lungs, and urinary tract [ 1 ]. Risk factors for fungal infections include exposure to humid environments, weakened immune systems, and underlying medical conditions, like diabetes [ 2 ]. In Indonesia, as a tropical country with high humidity, the risk of skin fungal infections is increased [ 3 ].

*Candida*, a genus of yeast, is a leading cause of fungal infections, and candidiasis is found worldwide, affecting all ages and genders. *Candida albicans* is the most common species worldwide, representing a global average of 66% of all Candida species [ 4 ]. However, its incidence varies geographically, ranging from 37% in Latin America to 70% in Norway [ 5 ]. In Asia, *C. albicans* is also prevalent, accounting for 56% of candidiasis cases in Hong Kong, and 33.3-55.6% of *Candida* bloodstream infections in Singapore, Taiwan, and Japan. Nevertheless, other *Candida* species, such as *C. parapsilosis* and *C. tropicalis*, are emerging as significant pathogens in certain regions, like Thailand and Malaysia [ 6 ].

Type of fungus and natural history of the infection are determined by the underlying predisposing conditions of the host [ 7 ]. Ability of yeast to morph into hyphae is considered a primary pathogenic mechanism, and it has been demonstrated that hyphae adhere more strongly to epithelial surfaces. The yeast form is now known to be invasive and is no longer considered merely commensal [ 8 ]. 

Candidiasis is often misdiagnosed as dermatitis, leading to self-treatment and obscuring the true picture of the disease [ 9 ]. Establishment of an early diagnosis of systemic candidiasis can be challenging due to nonspecific clinical signs and frequently negative cultures. Furthermore, there is no definitive prophylactic regimen for high-risk patients [ 10 ].

Drug resistance is a serious global health issue, and antifungal resistance is increasing worldwide. It is predicted that nearly 3 million Americans will develop drug-resistant infections this year, with over 35,000 deaths expected [ 11 , 12 ]. In Indonesia, resistance to antifungal drugs, such as azoles and echinocandins, is also increasing, with some fungal species, like *Candida glabrata* and *Candida auris*, showing resistance to multiple classes of antifungal drugs. This resistance poses a serious problem, as it can lead to treatment failure and increased mortality rates in patients with invasive fungal infections [ 13 , 14 ].

Therefore, this study aimed to investigate trends in *Candida* species distribution and their susceptibility patterns to various antifungal agents. By understanding the local epidemiology and antifungal resistance patterns, we can develop effective strategies to manage *Candida* infections and improve patient outcomes. With the acquired data, *Candida* species infection cases are expected to be managed more effectively.

## Materials and Methods

This study was conducted at the Clinical Microbiology Laboratory of the University of Indonesia on 2,470 specimens that were obtained during 2020-2024. A variety of specimens were collected from patients suspected of having mycosis. Demographic information of patients was obtained from their medical records. This study used retrospective secondary data from WHONET 2022, initially collected for antimicrobial resistance surveillance. There was no direct participant contact, and the data contained no identifiable information; hence, no risks to privacy, confidentiality, or participant safety. Accordingly, ethical approval was not required from an institutional review board or ethics committee.

### Specimen collection

Specimens were collected and processed in accordance with established protocols, with immediate transportation to the laboratory for analysis.

### Culture, identification, and susceptibility test

Another specimen, such as body fluid, is inoculated on a BacT/Alert (BioMerieux Inc., France) bottle and incubated on a BacT/Alert, an automated microbial detection system based on the colorimetric detection of CO2 produced by growing microorganisms [ 15 ].

The plates on Sabouraud Dextrose Agar (Oxoid, United Kingdom) were incubated at 30°C (Thermo) and observed every day. Yeast isolates were identified by Gram staining (Becton Dickinson, USA) and Lactophenol Cotton Blue (LPCB) (Himedia, India) [ 16 ].

For identification and susceptibility testing, the Vitek 2® compact automated system with YST ID and AST-YS01 cards was utilized (BioMérieux Inc., France). Inoculum turbidity was standardized using a DensiCHEK instrument, targeting a range of 1.8-2.2 McFarland units (BioMérieux, Inc., France) [ 17 ].

Two sterile 12 × 75 mm polystyrene tubes (Tube I for identification and Tube II for susceptibility testing) are provided, each filled with 3 ml of sterile 0.45% NaCl solution (pH 5.0) and placed in a cassette. The fungal suspension in Tube I is standardized. Then, 280 μl of the suspension is transferred from tube I to tube II, which is placed in the AST card cassette parallel to tube II. The cassette is then loaded into the Vitek instrument for processing. The incubation process takes several hours, and the results are printed automatically [ 17 ].

Sensitivity in the context of microbiology and pharmacology refers to the susceptibility or responsiveness of a microorganism to an antimicrobial agent, such as an antifungal. Antifungal sensitivity refers to the ability of an antifungal to inhibit or kill a specific microorganism. The breakpoint for each MIC value of antifungals against *Candida* species is based on the reference from the Clinical and Laboratory Standards Institute (CLSI) [ 18 ].

## Results

Over the period 2020-2024, 799 (32.34%) of 2,470 samples tested positive for pathogenic fungi. Specimens were received in the microbiology laboratory for yeast culture from patients with various infections from 44 hospitals, 11 clinical laboratories, and 10 independent medical doctors in Jakarta and its surrounding areas. 

The proportions of gender between male and female were 420 (55.57%) and 379 (47.43%). The age of patients ranged from 17 days to 98 years, with the highest prevalence observed in the 70-79 years age group (21.02%) (Table 1).

**Table 1:** Demographic data of patients

Variable	Number (%)	
Gender	Male	420 (52.57)
	Female	379 (47.43)
Age range(years)	<1	9 (1.12)
	1-9	12 (1.5)
	10-19	15 (1.87
	20-29	29 (3.63)
	30-39	67 (8.39)
	40-49	67 (8.39)
	50-59	124 (15.52)
	60-69	166 (20.78)
	70-79	168 (21.02)
	80-89	122 (15.26)
	90-99	20 (2.5)
Clinical specimen	Sputum	440 (55.01)
	Urine	131 (16.39)
	Broncho alveolar lavage	97 (12.14)
	Blood	40 (5.00)
	Faeces	29 (3.63)
	Vaginal swab	15 (1.88)
	Nail scrapping	8 (1.00)
	Tissue	7 (0.87)
	Isolate	7 (0.87)
	Abscess	6 (0.75)
	Swab throat	4 (0.50)
	Swab ears	3 (0.37)
	Endocervical swab	3 (0.37)
	Hair	2 (0.25)
	Vitreous fluid	2 (0.25)
	Ascitic fluid	1 (0.13)
	Pleural fluid	1 (0.13)
	Nasopharyngeal tube	1 (0.13)

The clinical specimens consisted of 20 types, with the 5 most common being sputum (n=440, 55.01%), urine (n=131, 16.39%), bronchoalveolar lavage (n=97, 12.14%), blood (n=40, 4.00%), and faeces (n=29, 3.63%) (Table 1). 

A total of 799 *Candida* isolates were identified, consisting of 21 species. The most *Candida* found were *C. albicans* (44.68%), *C. glabrata* (17.02%), and *C. tropicalis* (16.15%). Other species included *C. krusei* (2.88%), *C. ciferrii* (2.00%), *C. laurentii* (1.88%), and *C. famata* and *C. lusitaniae* (0.75%), respectively. Moreover, other fungi included *C. colliculosa* (0.38%), as well as *C. magnoliae*, *C. pelliculosa*, *C. rugosa*, and *C. sphaerica* (0.25%). Additionally, fungi detected at a percentage of 0.13% were *C. intermedia*, *C. kefyr*, *C. lipolytica*, *C. utilis*, and *C. haemulonii*.

Patterns of sensitivity of *Candida* species to various antifungals, such as fluconazole, voriconazole, caspofungin, micafungin, amphotericin B, and flucytosine, are presented in Table 2. The result indicated that the predominant *Candida* species exhibited high susceptibility (> 90%) to fluconazole, voriconazole, micafungin, amphotericin B, and flucytosine. Meanwhile, voriconazole, amphotericin B, and flucytosine showed excellent activity against uncommon *Candida* species, such as *C. lusitaniae*, *C. pelliculosa*, *C. rugosa*, *C. intermedia*, *C. kefyr*, *C. lipolytica*, and *C. utilis*. 

**Table 2:** Pattern of sensitivity of *Candida* species to various antifungals

*Candida* species	Antifungal (%)					
	Fluconazole	Voriconazole	Caspofungin	Micafungin	Amphotericin B	Flucytosine
*C. albicans*	348 (97.48)	341 (95.52)	350 (98.04)	348 (97.48)	339 (94.96)	351 (98.32)
*C. glabrataL*	136 (100)	123 (90.44)	23 (16.91)	129 (94.86)	128 (94.12)	134 (98.53)
*C. tropicalis*	117 (90.70)	122 (94.57)	122 (94.57)	122 (94.57)	124 (96.12)	123 (95.35)
*C. parapsilosis*	57 (91.94)	59 (95.16)	59 (95.16)	60 (96.77)	60 (96.77)	1 (1.61)
*C. dubliniensis*	8 (27.58)	25 (86.21)	1 (3.45)	-	26 (89.66)	26 (89.66)
*C. krusei*	23 (100)	22 (95.65)	18 (78.26)	23 (100)	20 (89.95)	3 (13.04)
*C. ciferii*	-	11 (68.75)	-	-	12 (75.00)	-
*C. guilliermondii*	-	3 (60.00)	3 (60.00)	3 (60.00)	3 (60.00)	4 (80.00)
*C. lusitaniae*	-	5 (100)	-	-	5 (100)	5 (100)
*C. pelliculosa*	-	1 (50.00)	-	-	1 (50.00)	1 (50.00)
*C. rugosa*	-	2 (100)	-	-	2 (100)	2 (100)
*C. sphaerica*	-	-	-	-	-	-
*C. intermedia*	-	1 (100)	-	-	1 (100)	1 (100)
*C. kefyr*	-	1 (100)	-	-	1 (100)	1 (100)
*C. 1ipolytica*	-	1 (100)	-	-	1 (100)	1 (100)
*C. utilis*	-	1 (100)	-	-	1 (100)	1(100)
*C. haemolonii*	-	-	-	-	-	-
*C. laurentii*	-	-	-	-	-	-
*C. famata*	-	-	-	-	-	-
*C. colliculosa*	-	-	-	-	-	-
*C. magnoliae*	-	-	-	-	-	-

Meanwhile, voriconazole and amphotericin B demonstrated over 60% susceptibility against *C. ciferrii*, while *C. guilliermondii* exhibited greater than 60% sensitivity to all four antifungal agents tested. *Candida* dubliniensis showed a favorable response to most antifungals, except fluconazole and caspofungin.

It is noteworthy that for several uncommon *Candida* species identified, such as *C. laurentii*, *C. famata*, *C. colliculosa*, *C. magnoliae*, *C. sphaerica*, and *C. haemulonii*, antifungal susceptibility data were not available. 

**Figure 1:**
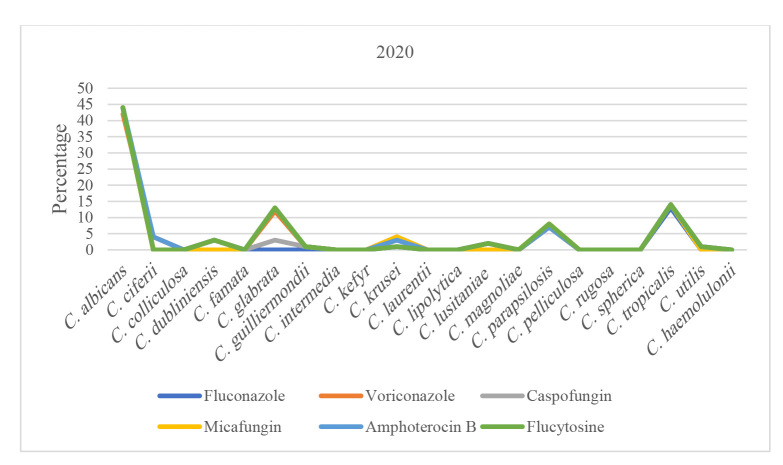
Susceptibility patterns of *Candida* species to antifungals in 2020.

**Figure 2:**
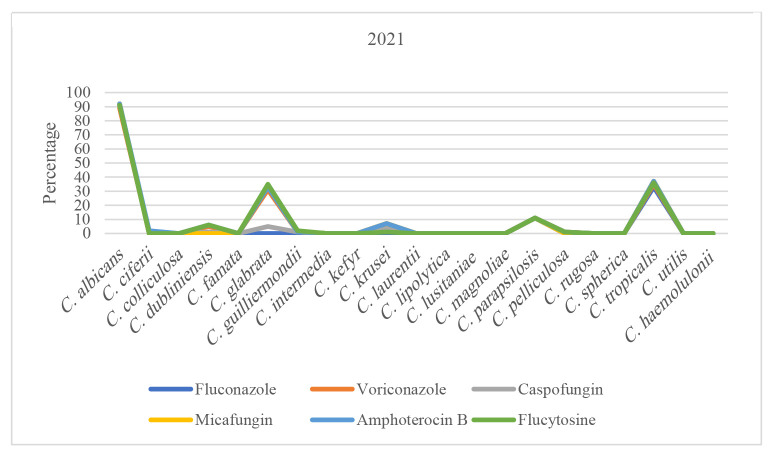
Susceptibility patterns of *Candida* species to antifungals in 2021.

**Figure 3:**
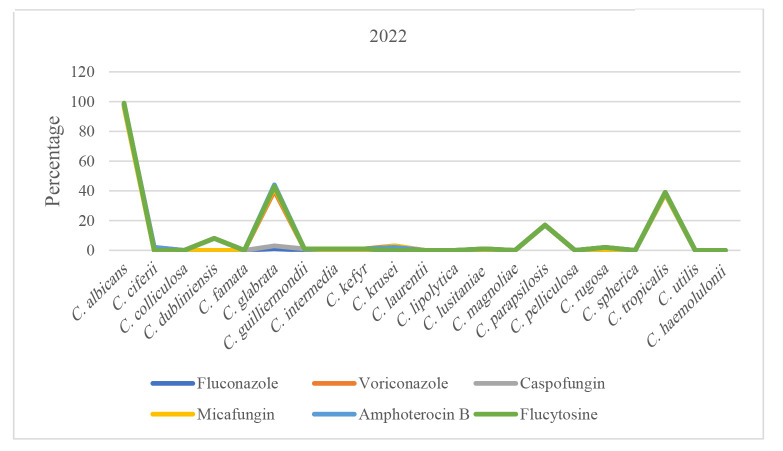
Susceptibility patterns of *Candida* species to antifungals in 2022.

**Figure 4:**
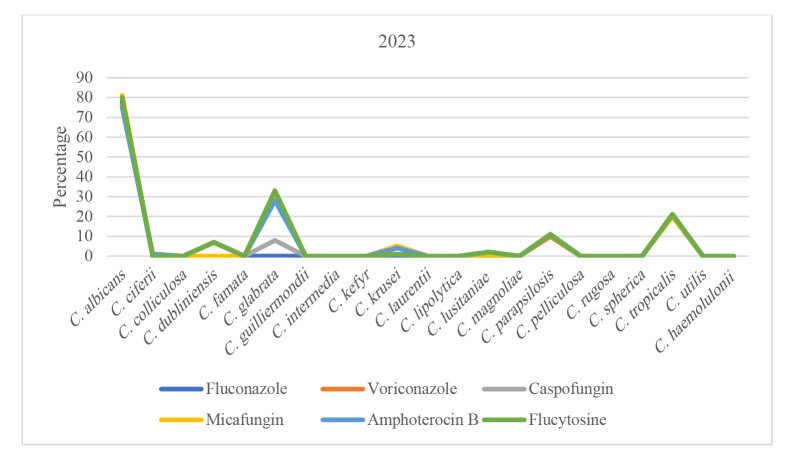
Susceptibility patterns of *Candida* species to antifungals in 2023.

**Figure 5:**
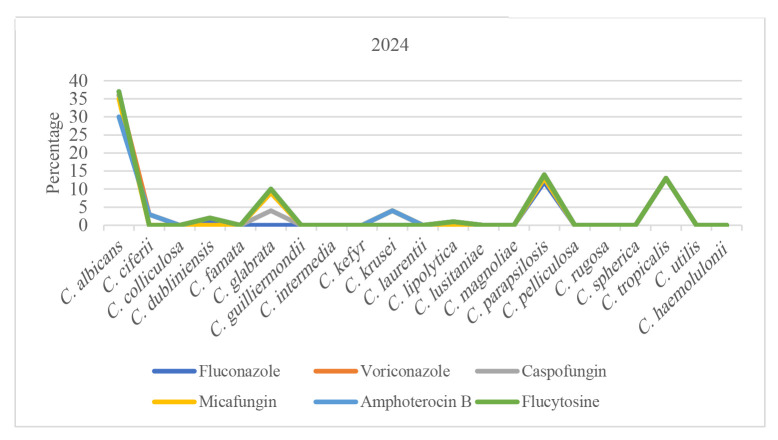
Susceptibility patterns of *Candida* species to antifungals in 2024.

Trend of antifungal susceptibility in *Candida* species over time can be seen in Figures 1 to 5.

## Discussion

The research data showed prevalence rates of 52.57% and 44.43% in males and females, respectively. These findings were consistent with those of studies performed by Conny et al. (2022) and Sano et al. (2021), which showed that the prevalence was higher in males, compared to females [ 19 , 20 ]. The higher prevalence of fungal infections in males, compared to females, may be related to several factors, including differences in immune response and risk factors. Research suggests that innate immune responses in females are stronger due to the regulation of the immune system by sex hormones. Additionally, factors such as poor personal hygiene, lack of circumcision, and long-term antibiotic use may increase the risk of fungal infections in men [ 20 , 21 ].

In the present study, the age range of patients was between less than 1 year and 98 years, with the majority falling within the 70-79 year age range (21.02%). These findings were consistent with those of the research conducted by Vidya et al. (2022) and Saniya (2024). The elderly are more susceptible to fungal infections due to several factors related to the aging process and changes in bodily functions, such as a weakened immune system. Moreover, skin changes, such as dryness and decreased sweat production, can increase the risk of infection. In addition, the presence of chronic diseases common among the elderly can compromise the resistance of body to infection [ 21 , 22 ]. 

The most commonly found specimens in the present study were sputum samples (55.01%). Sputum specimens are mostly found in fungal infections in patients with opportunistic fungal infections, particularly those with weakened immune systems or chronic diseases. Fungal species, like *Aspergillus* and *Candida*, are frequently found in the sputum of patients with these conditions. 

Consistent with the present study, *C. albicans* is a common fungus found in sputum [ 21 , 23 ]. When collected properly and in line with clinical symptoms, sputum samples can be a trustworthy specimen for diagnosing fungal infections. However, in the present study, patient data were highly limited, lacking key information, such as medical history, disease severity, and prior treatments. 

This study showed that the highest prevalence of *Candida* species was found to be *C. albicans*, *C. glabrata*, and *C. tropicalis*. Globally, *C. albicans* remains the most prevalent *Candida* species, while the second and third most common species vary between *C. parapsilosis*, *C. glabrata*, and *C. tropicalis* [ 24 - 26 ]. 

An interesting finding in the present study was the discovery of rare *Candida* species, namely, *C. krusei*, *C. ciferii*, *C. laurentii*, *C. famata*, *C. guilliermondii*, *C. lusitaniae*, *C. colliculosa*, *C. magnolia*, *C. pelliculosa*, *C. rugosa*, *C. sphaerica*, *C. intermedia*, *C. kefyr*, *C. 1ipolytica*, *C. utilis*, and *C. haemolonii*. Emergence of these uncommon *Candida* species highlights the need for increased awareness and vigilance, given their potential to cause significant health issues and difficulties in management [ 27 - 29 ]. 

*Candida haemulonii*, one of the rare *Candida* species, is a virulent pathogen that requires attention due to its resistance to antifungal agents and potential to cause chronic infections, particularly in diabetic patients [ 30 ]. 

The present study showed antifungal susceptibility results against predominant *Candida* species, in the following order: fluconazole (96.06%), voriconazole (93.51%), caspofungin (69.84%), micafungin (95.64%), amphotericin B (95.07%), and flucytosine (97.40%). These results are consistent with those of the studies performed by Conny et al. (2022) and Derek et al. (2024) [ 19 , 24 ].

*Candida parapsilosis* had low susceptibility (1.61%) to flucytosine but desirable susceptibility (90%) to other antifungals. These findings differ from those of Derek et al. (2024), who reported 100% susceptibility to fluconazole and flucytosine. The low susceptibility to these antifungals warrants caution and careful consideration in use [ 19 , 24 ]. 

*Candida dubliniensis* showed high susceptibility (> 80%) to voriconazole, amphotericin B, and flucytosine, but lower susceptibility to fluconazole (27.58%) and caspofungin (3.45%). There were no results from micafungin. *Candida krusei* was highly susceptible to fluconazole and micafungin (100%), moderately susceptible to voriconazole (95.65%) and amphotericin B (89.95%), less susceptible to caspofungin (78.26%), and had low susceptibility to flucytosine (13.04%). In contrast to a study performed by Nurulet al. [2018], who reported 100% susceptibility to both fluconazole and flucytosine, the present study found a discrepancy in the susceptibility rate to flucytosine [ 31 ]. 

Among the rare *Candida* species, *C. guilliermondii* showed the highest susceptibility, with over 60% susceptibility to voriconazole, caspofungin, micafungin, amphotericin B, and flucytosine; however, there was no data on fluconazole.

In contrast, other species, such as *C. lusitaniae*, *C. pelliculosa*, and *C. rugosa*, as well as *C. intermedia*, *C. kefyr*, *C. lipolytica*, and *C. utilis*, showed 100% susceptibility to voriconazole, amphotericin B, and flucytosine. However, there was no data on fluconazole, caspofungin, and micafungin. Meanwhile, *C. ciferrii* was susceptible (> 60%) only to voriconazole and amphotericin B, consistent with the findings of Aynaz et al. (2024), who reported amphotericin B to be effective [ 32 , 33 ].

One of the concerning findings in this study was the discovery of uncommon *Candida* species, such as *C. laurentii*, *C. famata*, *C. colliculosa*, *C. magnoliae*, *C. sphaerica*, and *C. haemulonii*. However, there were no data on their susceptibility to various antifungals. This is particularly worrisome given that these rare fungal species have developed resistance to multiple antifungals. Several studies have reported that *C. haemulonii* exhibits resistance to multiple antifungals, and its ability to form biofilms is a key virulence factor contributing to its persistence and difficulty in eradication [ 34 , 35 ]. 

The inability to obtain antifungal susceptibility results for certain species in the Vitek test may be due to several factors, including limited database coverage, interspecies variability, and methodological limitations. [ 36 ]. 

Findings of the present research differ from those of previous ones, which have reported higher susceptibility rates, including 100% effectiveness of fluconazole, voriconazole, and amphotericin B, followed by flucytosine (91.66%), micafungin (86.18%), and caspofungin (85.74%) [ 32 - 35 ]. 

An analysis of antifungal susceptibility trends over five years revealed noteworthy patterns among the predominant *Candida* species. In 2020, *Candida C. albicans*, *Candida C. glabrata*, and *Candida C. tropicalis* demonstrated average susceptibilities of 99.25%, 69.23%, and 98.79%, respectively.

In 2021, the susceptibility rates for *C. albicans* and *C. tropicalis* remained relatively stable. However, *C. glabrata* exhibited a modest decline in susceptibility, decreasing to 65.71%. During the subsequent years, 2022 and 2023, susceptibility patterns across all three species remained largely consistent with those of prior observations.

*Candida glabrata* susceptibility rose to 70% in 2024. This increase was due to antimicrobial stewardship, infection prevention, and bacterial ecology, beyond just biology [ 36 ]. 

In 2024, other *Candida* species (*C. parapsilosis*, *C. dubliniensis*, *C. krusei*, and *C. ciferrii*) had varying susceptibility trends; accordingly, *Candida parapsilosis* stayed above 90%, *C. dubliniensis* went from 60% (2020-2022) to 80% (2023), and then dropped to 70%. Moreover, *Candida krusei* susceptibility was stable at > 60% (2020-2022), dipped to 54.18% (2023), then rose to 66.75% (2024). In addition, *C. ciferrii* susceptibility to voriconazole and amphotericin B increased from 50% (2020) to 100% (2021-2024).

*Candida guilliermondii* susceptibility varied, with 100% sensitivity in 2020 and 2022, dropping to 62.5% in 2021, and resistance emerging in 2023-2024. This requires vigilance, despite average *Candida* susceptibility rates being 60-80%.

*Candida lusitaniae* showed 100% sensitivity to antifungal agents in 2020, 2022, and 2023, with no resistance detected in 2021 and 2024, indicating sustained susceptibility. *Candida pelliculosa* and *C. rugosa* had 100% susceptibility in 2021-2022. Rare species like *C. intermedia*, *C. kefyr*, *C. utilis*, and *C. lipolytica*, isolated in single years, also showed 100% susceptibility. 

Declining *Candida* susceptibility is concerning, fueled by rare species emergence, reducing antifungal effectiveness. Fungi resist drugs via target mutations, efflux pumps, structural changes, and metabolic adaptations [ 36 , 37 ]. Several key factors contribute to fungal resistance to antifungals, including inappropriate antifungal use, excessive antibiotic use, agricultural and industrial fungicide use, and host-related factors, such as health conditions and immune status [ 36 , 37 ].

Management of antimicrobial resistance involves prudent use, proper dosing, specific antimicrobials, resistance monitoring, new drug development, infection prevention, and education. A comprehensive approach is key to addressing antifungal resistance.

Based on the data obtained in this study, recommended empirical therapy for *Candida* infections is flucytosine, fluconazole, and micafungin for the predominant species. Addressing fungal resistance requires effective treatments and judicious antifungal use.

## Conclusion

This study found *C. albicans*, *C. glabrata*, and *C. tropicalis* as the most common *Candida* species. They showed high susceptibility to flucytosine, fluconazole, and micafungin. Continuous local surveillance is crucial to guide antifungal treatment due to variability in species distribution and susceptibility over time.
